# Supplementary material for: Content-rich biological network constructed by mining PubMed abstracts
Source: BMC Bioinformatics. 2004 Oct 8;5:147. doi: 10.1186/1471-2105-5-147 (PMC528731; doi:10.1186/1471-2105-5-147)
Supplement: Additional File 2 — The original results of the above study (non-essential files are deleted to keep the file size under the limit set by BMC bioinformatics). [file 1471-2105-5-147-S2.bz2 › chilibotAdditionalFile2/dip30/645ID11060015E11032/html/CRR_PTSH.html]

 


 **CRR** and **PTSH** 
  
Found 24 abstracts in PubMed, retrieved 24.  
 

 What does Google say? 
 PDF only 
| .edu only 

---

**Interactive relationship** (e.g. stimulation, inhibition, etc)

**Neutral relationship**- This gene, unlike the E. coli  **crr**  gene, was located to the left of  **ptsH** .  Ref: 3106335 J Bacteriol, 1987
- The  **ptsH**  gene is transcribed in the same direction as the ptsI gene and the  **crr**  gene encoding enzyme IIIGlc of the PTS.  Ref: 2411636 Gene, 1985

**Non-interactive relationship** (e.g. studied together, co-existance, homology, etc.)

- Analysis of the nucleotide sequence substantiates the notion that the  **ptsH**  ptsI  **crr**  genes constitute a polycistronic operon.  Ref: 2411636 Gene, 1985
- The present study showed that i the  **ptsH** , ptsI, and  **crr**  genes exhibited high basal expression, ii transcription of the  **ptsH**  and ptsI genes was stimulated threefold by the cyclic AMP cyclic AMP receptor protein complex and also by growth on glucose, but only in the presence of an active enzyme IIGlc, iii  **crr**  specific expression was not sensitive to the complex or to growth on glucose, and iv under the growth conditions tested, the major part of  **crr**  transcription was initiated from internal promoters.  Ref: 2457575 J Bacteriol, 1988
- The cloning of the PTS region as a whole allowed us to map and order  **ptsH** , ptsI and  **crr**  genes in a 3.8 kb DNA fragment and determine that ptsI and  **crr**  are transcribed from a common promoter.  Ref: 6099314 Gene, 1984
- Analysis of the  **ptsH**  ptsI  **crr**  region in Escherichia coli K 12 nucleotide sequence of the  **ptsH**  gene.  Ref: 2411636 Gene, 1985
- A cosmid containing the  **ptsH** , ptsI and  **crr**  genes of Escherichia coli coding for three components of the phosphoenolpyruvate sugar phosphotransferase system PTS has been isolated.  Ref: 6099314 Gene, 1984
- Analysis of the  **ptsH**  ptsI  **crr**  region in Escherichia coli K 12 evidence for the existence of a single transcriptional unit.  Ref: 6099314 Gene, 1984
- The results give a gene order of cysA cysM  **crr**  ptsl  **ptsH**  cysK over a distance of approximately 12 kilobases.  Ref: 3531173 J Bacteriol, 1986
- Expression plasmids were constructed which contained the genes encoding enzyme I ptsI, HP  **ptsH** , IIAGlc  **crr** , and IICBGlc ptsG of the glucose phosphotransferase system behind inducible promoters.  Ref: 7601098 Eur J Biochem, 1995
- Specialized lambda transducing phages that carry the Escherichia coli genes  **ptsH** , ptsI,  **crr** , cysM, and cysA have been isolated, and the genes were subcloned in plasmid pBR322.  Ref: 2960675 J Biol Chem, 1987
- In glucose minimal medium a PTS strain of Escherichia coli delta  **ptsH**  ptsI  **crr**  could grow slowly doubling time, d = 10 h.  Ref: 1659611 J Gen Microbiol, 1991
- In both  **ptsH** , I, and  **crr**  mutants the amount of galactose binding protein, one of the components of MglP, is only 5% 20% of that in wild type cells, as measured with a specific antibody.  Ref: 6267419 Mol Gen Genet, 1981
- In double label experiments, the vesicle volumes were found to be about 6 microliters mg of protein for preparations isolated from the wild type strain, and about 4.5 microliters mg of protein for vesicles isolated from a mutant, SB2950, deleted in  **ptsH** , ptsI, and  **crr**  genes proteins HPr, Enzyme I, and IIIGlc, respectively.  Ref: 6754736 J Biol Chem, 1982
- The nucleotide sequence of a 1689bp fragment of the Bacillus subtilis locus containing ptsX a  **crr**  like gene,  **ptsH**  coding for HPr, and the 5 end of ptsI coding for Enzyme I was determined.  Ref: 2497294 Mol Microbiol, 1989
- Molecular cloning and structural analysis of the Escherichia coli  **ptsH** , ptsI, and  **crr**  genes.  Ref: 2960675 J Biol Chem, 1987
- One encompassed the three cistrons, a second one the  **ptsH**  gene and part of the ptsI gene, and the third one only the distal gene  **crr** .  Ref: 2457575 J Bacteriol, 1988
- In contrast, we successfully identified three phosphotransferase system genes,  **ptsH** , ptsI, and  **crr**  in Buchnera, and provide evidence for their active expression.  Ref: 10467175 J Biochem Tokyo , 1999
- The pts operon of Escherichia coli is composed of the genes  **ptsH** , ptsI, and  **crr** , which code for three proteins of the phosphoenolpyruvate dependent phosphotransferase system PTS the HPr, enzyme I EI, and EIIIGlc proteins, respectively.  Ref: 1898933 J Bacteriol, 1991
- The three genes constitute an operon, but analysis of the  **ptsH** , ptsI, and  **crr**  transcripts by Northern RNA blotting revealed the existence of three major mRNA species.  Ref: 2457575 J Bacteriol, 1988
- Three genes encoding homologues of the general PTS components enzyme I ptsI, HPr  **ptsH** , and enzyme IIA  **Crr**   **crr** .  Ref: 11386346 Antonie Van Leeuwenhoek, 2000
- The  **ptsH** , ptsI, and  **crr**  genes, coding for three of the proteins of the phosphoenolpyruvate dependent phosphotransferase system PTS HPr, enzyme I, and enzyme IIIGlc, respectively have been studied by determination of their nucleotide sequence and analysis of their expression.  Ref: 2457575 J Bacteriol, 1988
- The pts operon of Escherichia coli is composed of the  **ptsH** , ptsI and  **crr**  genes coding for three proteins central to the phosphoenolpyruvate dependent phosphotransferase system PTS, the HPr, enzyme I and EIIIGlc proteins, respectively.  Ref: 1324322 J Mol Biol, 1992
- The  **ptsH** , ptsI, and  **crr**  genes of the Escherichia coli phosphoenolpyruvate dependent phosphotransferase system a complex operon with several modes of transcription.  Ref: 2457575 J Bacteriol, 1988
- Phosphoenolpyruvate sugar phosphotransferase system of Bacillus subtilis cloning of the region containing the  **ptsH**  and ptsI genes and evidence for a  **crr**  like gene.  Ref: 3106335 J Bacteriol, 1987
- A model of regulation for the  **ptsH**  ptsI  **crr**  operon transcription is presented which accounts for antisense transcription.  Ref: 2469621 FEMS Microbiol Lett, 1989
- The nucleotide sequences of  **ptsH** , ptsI, and  **crr**  and the corresponding flanking regions have been determined.  Ref: 2960675 J Biol Chem, 1987
- Comparison of the physical and linkage maps of the E. coli chromosome in the 52 minute region has permitted precise mapping of most of the 18 genes in this region with the order nupC glk less than alaW beta ala W alpha 1 kb less than gltX 0.3 kb valU alpha valU beta valU gamma lysV = supN greater than xapR xapA less than lig 1 kb cysK greater than 0.4 kb  **ptsH**  greater than 0.05 kb pstI greater than 0.05 kb  **crr**  greater than cysM cysA in the clockwise order greater than and less than indicate the direction of transcription.  Ref: 2201776 J Mol Biol, 1990
- Subcloning and restriction mapping data gave the following clockwise order of genes located at about 52 min on the E. coli genetic map lig, cysK,  **ptsH** , ptsI,  **crr** , cysM, cysA.  Ref: 2960675 J Biol Chem, 1987
- BACKGROUND The pts operon of Escherichia coli consists of three genes  **ptsH** , ptsI and  **crr** , each encoding for central components of the phosphoenolpyruvate carbohydrate phosphotransferase system, HPr, enzyme I and IIAGlc, respectively.  Ref: 10469172 Genes Cells, 1999
- Expression of the genes was studied in vivo by using operon and protein fusions between the lacZ gene and the  **ptsH** , ptsI, or  **crr**  gene on IncW low copy number plasmids.  Ref: 2457575 J Bacteriol, 1988
